# Successful Myomectomy during Cesarean Section: Case Report & Literature Review

**Published:** 2017-06

**Authors:** Fatemeh Ghaemmaghami, Mojgan Karimi-Zarchi, Mahin Gharebaghian, Tahere Kermani

**Affiliations:** 1 Professor of gynecology, Tehran University of medical sciences, Iran;; 2 Gynecologic Oncology fellowship, Shahid Sadoughi University of Medical Science, Yazd, Iran;; 3 Associate professor of anesthesiology, Tehran university of medical sciences, Iran;; 4 Gynecologist & Obstetrician, Mehr General Hospital, Tehran, Iran

**Keywords:** Myoma, Fibroma, Leiomyoma, Myomectomy, pregnancy, Cesarean section

## Abstract

Myomectomy is the most common surgery with cesarean section. There is controversy between obstetricians about doing myomectomy with cesarean section. A 29 years old primigravida patient presented with a large lower segment myoma (20 cm.) who underwent myomectomy during cesarean section at the term pregnancy. Myoma weighted 1500 gr. She didn't have intra-operative hemorrhage or any post-partum complications. Seems that there is no absolute contra-indication for myomectomy during cesarean section specially if the surgeon has enough experience and the myoma is large, located at the lower segments.

## INTRODUCTION

Myomectomy is the most common surgical operation performed during cesarean section and in a study that has reviewed 10 years of experience, it was about 0.89% of all cesarean sections (1). But most obstetricians are trained to avoid removal of large myomas during cesarean section before last decade.

Pedunculated myomas can easily be removed and hemostasis can be secured at the same time without endangering the mother’s life.

Myomectomy during cesarean section was practically absent from the obstetrics literature until the last decade and it was suggested to post pone the myomectomy and perform it perhaps before the next pregnancy (2).

The most common reason of this suggestion has been preventing from unwanted hysterectomy because of excessive uncontrollable hemorrhage during myomectomy (2-4).

Some obstetricians, however, believe that with careful case selction, surgical management of uterine myomas during cesarean section maybe safe (3, 5).

For example when the myomas are located at the lower segment of the uterus, the obstetricians may prefer the classical route (2). Successful inevitable myomectomy during cesarean section has been reported by a few of authors (2, 6, 7).

## CASE REPORT

**Figure 1:**
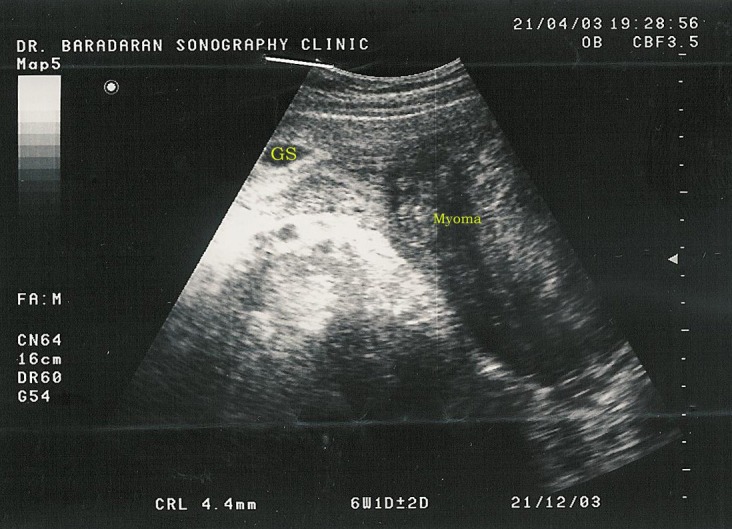
Uterine Sonography in 6 weeks of pregnancy.

A 29 years old primigravida patient presented with a 6 weeks history of amenorrhea. In physical examination she had a uterus of 6 weeks pregnancy and the ultrasonography examination of the abdomen showed a myoma measuring about 107 × 60 mm (Fig. 1).

She didn’t have any symptoms related to the myoma such as abnormal uterine bleeding or pain, before pregnancy and also there were no complications during pregnancy due of myoma. For example the level of her hemoglobin before and after operation was 13.4 and 12.8 mg/dl.

In serial sonographic studies, the size of the myoma was increasing and in the last one, the weight of fetus was below 10% of normal growth curve. At the third trimester, the myoma was palpable, about 15-20 cm. so she was considered to undergo the elective cesarean section due to the probability of obstructed labor in 38-39 weeks of pregnancy.

After consultation with anesthesiologist, the cesarean section was performed.

The abdomen was opened with pfannenstiel incision. After take down of the bladder the lower segment of the uterus was incised and due to the huge myoma in the lower segment, the incision was extended a bit upper. When the amniotic membrane was found and ruptured, a female 2500 gr. weighted newborn was delivered with difficulty. The apgar score of the newborn was 8 at the first minute.

After removal of the placenta high dose oxytocin (30 IU /1000 cc. ringer lactate) was infused in 1 hour.

The myomectomy was done and a single intra-mural myoma measuring about 20 cm. weighting 1500 gr. was removed. During the procedure of myomectomy, infusion of oxytocin was continued.

The amount of hemorrhage was almost 1200 cc that was similar to other cesarean section cases which was compensated with ringer-lactate solution.

The duration of operation was 38 minute that added 10 minutes and Hgb. level after surgery was 12.8.

**Figure 2:**
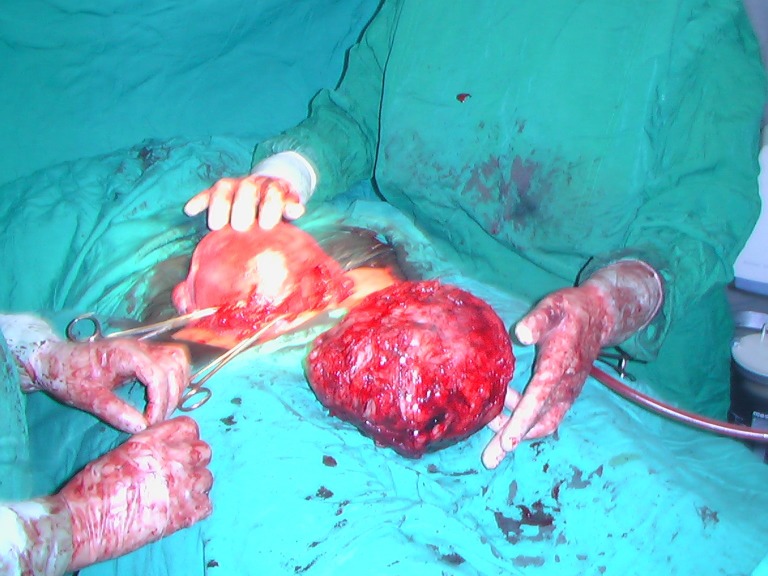
Uterus after cesarean section & removed myoma.

The suture line over the uterus after the surgery, was completely similar to those cases of lower segment cesarean section (Fig. 2).

The histological examinations confirmed the diagnosis of myoma.

The patient was discharged two days after surgery and at the time of discharge, involution of uterus was normal and we didn’t face any post partum morbidity.

## DISCUSSION

This case showed that myomectomy during cesarean section may not be as dangerous generations of obstetricians and gynecologists have been trained to believe.

With large myomas in lower segment of the uterus, myomectomy may be inevitable and there appears to be no absolute contra-indication to myomectomy.

Whereas small fibroid <2-3 cm and single, myomectomy during cesarean section probably is not indicated especially when it is asymptomatic.

With an adequate experience in myomectomy during cesarean section and use of high dose oxytocin infusion, severe hemorrhage which is the most serious complication can be controlled.

Omar SZ, *et al*. (7) reported 2 cases of large uterine myomas situated in the anterior aspect of the lower segment, complicating pregnancy at term, myomectomy in both instances allowed delivery of the fetus through the lower segment.

From review literature is seen that Douglas and stromme reported 13 cases of myomectomy at cesarean section with one complicated by severe intra-operative hemorrhage.

Also of the nine cases of myomectomy during cesarean section that was reported by Exacousts and Rosat three were complicated by severe hemorrhage necessitating hysterectomy (8, 9). They concluded that the decision to perform hysterectomy during cesarean section should be made with caution because of risk of hemorrhage.

On the otherwise, other studies have showed a high incidence of hysterectomy for post-partum hemorrhage at delivery and puerperium period and post-partum sepsis (10, 11) in which the myoma was not removed. So removal of the myoma during cesarean section seems to be logical.

In a study done by Orac *et al*. (12), 22 patients with large myomas (>5 cm) underwent myomectomy during cesarean section, neither hysterectomy nor hypogastric artery ligation or any other procedure was needed to control hemorrhage. There was no perinatal death.

Burton *et al*. (13) reported 13 cases of incidental myomectomy at cesarean section, only one case was complicated by intra-operative hemorrhage attributable to the myomectomy. They suggested that myomectomy in cesarean section may be safe in carefully seleted patients.

In case control study done be kwawukume *et al*. (2) 12 patients with myomectomy during cesarean section, involution of the uterus was normal in all of the patients and there was no intra-operative hemorrhage significantly higher than control cases.

Another study (14) has showen that myomectomy as a separate operation during cesarean section increased the rate of hemorrhage by 10%.

Other studies showed (2, 15) that myomectomy during cesarean section adds the time of surgery by about 11 minutes which is similar to our study.

## CONCLUSION

It seems to be safe to perform myomectomy during cesarean section if surgeon is experienced and the size and location of myoma is considered.

It’s better to remove large myomas in lower segment because they can prohibit post-partum hemorrhage and sepsis but with small myomas on fundus of uterine, myomectomy may not be indicated (16-19).
